# Development of a Two-Stage Microalgae Dewatering Process – A Life Cycle Assessment Approach

**DOI:** 10.3389/fpls.2016.00113

**Published:** 2016-02-11

**Authors:** Rizwan R. Soomro, Theoneste Ndikubwimana, Xianhai Zeng, Yinghua Lu, Lu Lin, Michael K. Danquah

**Affiliations:** ^1^Department of Chemical Engineering, Faculty of Engineering and Science, Curtin University of TechnologySarawak, Malaysia; ^2^Department of Chemical and Biochemical Engineering, College of Chemistry and Chemical Engineering, Xiamen UniversityXiamen, China; ^3^College of Energy, Xiamen UniversityXiamen, China; ^4^The Key Laboratory for Synthetic Biotechnology of Xiamen City, Xiamen UniversityXiamen, China

**Keywords:** microalgae, dewatering, biomass, biofuels, bioprocess

## Abstract

Even though microalgal biomass is leading the third generation biofuel research, significant effort is required to establish an economically viable commercial-scale microalgal biofuel production system. Whilst a significant amount of work has been reported on large-scale cultivation of microalgae using photo-bioreactors and pond systems, research focus on establishing high performance downstream dewatering operations for large-scale processing under optimal economy is limited. The enormous amount of energy and associated cost required for dewatering large-volume microalgal cultures has been the primary hindrance to the development of the needed biomass quantity for industrial-scale microalgal biofuels production. The extremely dilute nature of large-volume microalgal suspension and the small size of microalgae cells in suspension create a significant processing cost during dewatering and this has raised major concerns towards the economic success of commercial-scale microalgal biofuel production as an alternative to conventional petroleum fuels. This article reports an effective framework to assess the performance of different dewatering technologies as the basis to establish an effective two-stage dewatering system. Bioflocculation coupled with tangential flow filtration (TFF) emerged a promising technique with total energy input of 0.041 kWh, 0.05 kg CO_2_ emissions and a cost of $ 0.0043 for producing 1 kg of microalgae biomass. A streamlined process for operational analysis of two-stage microalgae dewatering technique, encompassing energy input, carbon dioxide emission, and process cost, is presented.

## Introduction

The depletion of fossil resource reserves, climate change as well as the increasing price of crude oil are amongst the challenging problems of the world today, hence the search for alternative fuels is imperative (Shirvani et al., 2011). Biofuels, such as biodiesel and bioethanol, are considered as alternatives to fossil based fuels. They have much more advantages over fossil fuels as they have relatively low toxicity, high biodegradability, low net CO_2_, are renewable and sustainable, and contains less or no sulfur (Zheng et al., 2012). Biofuels are currently produced from biological materials including sugar, corn, vegetable oils, plants, animal fats, woody biomass, and bio-wastes. However, due to competition with human food chain and the significant pretreatment operational costs associated with woody biomass and bio-wastes, the search for new biomass sources has been a major research endeavor globally. Microalgal biomass is considered as an attractive feedstock for biofuel production due to its several significant advantages such as rapid growth rate, high lipid and carbohydrate contents, limited competition with food crops for arable land, and the great potential for carbon capture and biosequestration (Vandamme et al., 2011). However, high energy inputs and operational cost during the production of biomass are major limitations associated with microalgal biofuels development (Pienkos and Darzins, 2009; Wijffels and Barbosa, 2010). It has been reported that one of the most energy and cost intensive steps in algal biomass production process is the harvesting and dewatering or drying of microalgae suspension (Uduman et al., 2010; Rawat et al., 2013), this is due to the low concentration in the culture medium and the microalgae small cell sizes (a few micrometer) (Vandamme et al., 2011). Microalgal culture dewatering techniques commonly used are classified as chemical, mechanical, electrical, and biological. These methods can be applied as a single technique or combined (Danquah et al., 2009b). Chemical dewatering methods are mostly flocculation induced by inorganic or organic polyelectrolyte (polymer) flocculants. Mechanical techniques include centrifugation, filtration, natural sedimentation, flotation, and foam separation. Electrical dewatering techniques are based on electro-coagulation process. Biological dewatering techniques include auto-flocculation occurring at high pH, flocculation caused by secreted biopolymers, and microbial flocculation (Christenson and Sims, 2011). Though not fully developed for commercial application, biological dewatering techniques have recently gained research attention as low-cost sustainable techniques because of the absence of synthetic chemicals and minimal energy consumption (Christenson and Sims, 2011). It is imperative to find an efficient, cost effective and environmentally friendly harvesting and dewatering technique for commercializing biofuels from microalgal biomass. For biofuels production, the main objective of dewatering is to concentrate the dilute microalgae suspension of about 0.02–0.06% to 5–25%, and this is achievable in a two-step dewatering process where the primary stage is aimed at 2–7% and the secondary stage produces 15–25% of microalgae slurry (Uduman et al., 2010). However, with the introduction of innovative dewatering systems, the concentration of the slurry my exceed 25% in the secondary dewatering stage. The combination of two dewatering techniques has also been found to significantly improve the process by reducing the energy demands and/or total emissions (Khoo et al., 2011; Beach et al., 2012; Bilad et al., 2012; O’Connell et al., 2013; Weschler et al., 2014). The environmental impact analysis of the dewatering technique is a critical aspect in the assessment of microalgae as a potential biofuel feedstock. This is necessary to ensure reduced carbon footprint in the life cycle assessment (LCA) of the microalgal biofuel production process (Sander and Murthy, 2010; Uduman et al., 2010). The LCA illustrates the significance of dewatering to the scale up of microalgae-to-biofuels process engineering, hence there is a need for techno-economic and performance improvements to make microalgae biofuels less energy intensive and more commercially viable (Uduman et al., 2010). This study will assess the performances of a wide range of technologies currently applied for microalgae dewatering through a comparative study of key process parameters as a basis for the development of two-stage microalgae dewatering systems. The complete performance and economic assessments of the two-stage dewatering systems are presented with an attempt to determine the most viable two-step dewatering technique.

## Conventional Unit Technologies For Microalgae Dewatering

### Electrocoagulation

Electrocoagulation is described as environmentally friendly, highly selective, and potentially cost effective microalgae dewatering technique. **Table 1** presents some results on the performance and energy input of different electrocoagulation technologies reported as a means of microalgae dewatering. The main drawbacks associated with this technique are the cost of electricity, fouling of cathodes and periodic change of the anode materials (Uduman et al., 2011; Vandamme et al., 2011; Granados et al., 2012). The electrocoagulation process is influenced by many different factors such as the electrode material type, current density, temperature, pH, and any other coexisting ions (Gao et al., 2010). The electrocoagulation process is achieved by the combination of metal cations with negatively charged microalgal cells in the suspension carried towards the anode by electrophoretic motion (Mollah et al., 2004; Bukhari, 2008; Uduman et al., 2010). The amount of electricity which passes through the electrolytic solution determines the amount of metal dissolved into the solution (Mollah et al., 2004; Azarian et al., 2007; Bukhari, 2008).

**Table 1:** Performance and energy input of microalgae dewatering by electrocoagulation systems.

Electrocoagulation system	Process performance	Energy (kWh/m^3^)	Reference
Electrocoagulation–Flocculation	Microalgal recovery efficiency: 80–95%	0.15–1	Vandamme et al., 2011
Electrolytic flocculation	Not precised	0.331	Coward et al., 2013
Electrocoagulation and electroflotation	Microalgal recovery efficiency: 99%	0.3	Weschler et al., 2014

### Flocculation

Flocculation occurs once the solid particles in suspension collide and adhere to each other (Uduman et al., 2010). This dewatering process works well in separating algae cells from culture suspensions (Chen and Yeh, 2005; Knuckey et al., 2006; Henderson et al., 2008; Vandamme et al., 2010; Wyatt et al., 2012). The performance and energy input of some reported flocculation systems for microalgae dewatering are presented in **Table 2.** In the suspension, algae cells are stabilized by negative surface charge at wide pH values (Wyatt et al., 2012). Flocculation targets the development and sedimentation of flocs.

**Table 2:** Performance and energy input of microalgae dewatering by flocculation systems.

Mode of flocculation	Process performance	Energy (kWh/m^3^)	Reference
Alum flocculation	Harvesting efficiency: 99.01%	0.1	Weschler et al., 2014
Polymer flocculation in conjunction with Al_2_(SO4)_3_	Suspended solids (%) in concentrate: 15	14.81	Danquah et al., 2009a
FeCl_3_ – induced flocculation	Flocculation efficiencies: >90%	Not determined	Wyatt et al., 2012
Flocculation by Tanfloc and pH variation	Flocculation efficiencies: >96%	Not determined	Selesu et al., 2016

Theories on flocculation of microalgae have been described though some contradict (Schlesinger et al., 2012). Some of these theories state that flocculation occurs because the alkaline nature of the flocculants neutralizes surface charge of cells which could enable coalescence to larger flocs. Such theories are based on the assumption that electrostatic flocculation increases with increase in flocculant dosage in linear stoichiometric relations. This makes flocculation technology an expensive process as the performance hinges significantly on the amount of flocculant present. Contrary to the electrostatic flocculation theory, Schlesinger et al. (2012) proposed that the amount of alkaline flocculants is a function of the logarithm of microalgae cell density with dense culture requiring an order of magnitude less base than dilute suspensions at low flocculation pH values. The principle of how flocculants work is based on the premise that the microalgae particulates have identical surface charges which repel them from each other. However, Chen et al. (2013), achieved effective flocculation for harvesting the microalgal cells of *Scenedesmus* sp. by simply increasing the pH of the culture without introducing any flocculants. Different types and flocculant dosage influence both the extent and the rate of flocculation. Both parameters are critical in the flocculation process, therefore extensive study should be undertaken to determine optimal flocculants dosage and sedimentation time (Schlesinger et al., 2012). Initial microalgal biomass concentration is one of the factors that influence flocculation efficiency. It has been found that a linear relation exists between the dosage needed and initial microalgal biomass concentration. The amount of suspended microalgal cells increases with increasing biomass concentration, thus higher flocculants dosages are required to interact with the surface charges of the microalgal cells (Kim et al., 2011).

### Filtration

Filtration process consists of a permeable medium which restrains the movement of solid particles and allows liquid to pass through (Uduman et al., 2010). There are different types of filtration processes that are applicable for microalgae dewatering. These include magnetic filtration, vacuum filtration, pressure filtration, tangential flow filtration (TFF), and cross-flow filtration (Show et al., 2013). The performances and energy input of different filtration systems for microalgae dewatering have been studied extensively as depicted in **Table 3.** Filtration as a microalgae dewatering technology have many advantages. These include: high efficiency, low energy input (Danquah et al., 2009b), low cost, water recycle and reuse (Greenwell et al., 2010; Mata et al., 2010). However, it is generally difficult to filter biological feed because of the compressible nature of the biomass cake. The tendency of fouling with biological cells is high as biological feedstock may consist of mixtures of organic materials of different sizes, shapes and compressibility (Ríos et al., 2012). Microalgae cause significant fouling to filtration membranes through the release of extracellular organic matter which significantly increases the cake resistance and this is independent on the membrane material, however, it is negligible at low feed concentrations. It however increases exponentially with microalgae deposition rate. Also the effect of trans-membrane pressure on cake resistance indicates that microalgal cake deposit is compressible and fouling of membrane by microalgae is proportional to the amount of organic polymers released (Babel and Takizawa, 2010).

**Table 3:** The performance and energy input assessment of microalgae dewatering by filtration.

Filter type	Process performance	Energy (kWh/m^3^)	Reference
Natural filtration	Suspended solids (%) in concentrate: 1–6	0.4	Uduman et al., 2010
Pressure filtration	Suspended solids (%) in concentrate: 5–27	0.88	Uduman et al., 2010
Tangential flow filtration (TFF)	Suspended solids (%) in concentrate: 2.5–8.9	0.2-2.6	Danquah et al., 2009a,b; Weschler et al., 2014
Vibrating screen filter	Harvesting efficiency: 89%	0.4	Weschler et al., 2014
Chamber filter	Harvesting efficiency: 89%	0.88	Coward et al., 2013
Belt filter press	Harvesting efficiency: 89%	0.5	Weschler et al., 2014
Vacuum filters	Suspended solids (%) in concentrate: 18	5.9	Coward et al., 2013
Submerged microfiltration	Recovery efficiency: 98%	0.25	Bilad et al., 2012

### Magnetic Separation

The principle of magnetic separation is based on the fact that materials with differing magnetic moments experience different forces in the presence of magnetic field gradients, thus an externally applied field can be used to drive a selection process (Svoboda and Fujita, 2003; Yavuz et al., 2009). Due to its attractive advantages such as low cost, energy efficient and simple operation, high permeation fluxes, small land area utilization, no clogging and fouling problems, magnetic separation process has been used in microalgae removal from freshwater using functionalized magnetic particles (Gao et al., 2009; Liu et al., 2009; Bucak et al., 2011; Toh et al., 2012). The technology presents the main drawback of low adsorptive capacity, also the high cost of nano magnetic particles should be considered while deciding on the magnetic separation system (Franzreb et al., 2006; Liu et al., 2009). The performance of different magnetic separation systems as reported by different researchers is presented in **Table 4.** The energy consumption during magnetic separation was not determined, thus not presented in the **Table 4.** In a mixture with a magnetic component, the magnetic material could be easily separated with electro-magnets or permanent magnets. Adsorption occurs due to electrostatic attraction between the magnetic particles and microalgae cells, and this is affected by the stirring speed, pH of the suspension, magnetic particle dosage, hydrodynamic resistance, magnetic field strength and the flow rate (Xu et al., 2011). Medium concentration, pH and particle concentration are critical to achieving high separation efficiencies (Cerff et al., 2012). During their studies on harvesting microalgae cells by magnetic separation, Cerff et al. (2012) reported that the presence of cations and anions in the medium could increase the separation efficiency by five folds, however extensive studies should be undertaken on different media to substantiate the finding. Other research reports have established that lower pH and increase in magnetic particle dosage favor high recovery efficiency depending on the type of microalgae (Xu et al., 2011).

**Table 4:** The performance and energy input assessment of microalgae dewatering by magnetic separation.

Magnetic separation system	Algal cell removal efficiency (%)	Reference
High gradient and low gradient magnetic separation	>90	Toh et al., 2012
High gradient magnetic separation	>95	Cerff et al., 2012
*In situ* magnetic separation	>98	Xu et al., 2011
Magnetic polymer separation	99	Liu et al., 2009
Coagulation-magnetic separation	>99	Liu et al., 2013a

### Flotation

Flotation as a separation technique was firstly applied in mineral industry, and recently have been found effective for removing algae from suspension (Phoochinda and White, 2003; Csordas and Wang, 2004; Phoochinda et al., 2004; Wiley et al., 2009). Flotation have many advantages such as less energy consumption than centrifugation (Wiley et al., 2009; Coward et al., 2013), and can achieve high efficiency at short operation time (Coward et al., 2013).

Flotation process is induced by bubbles generated from air or gas transformation within a solid-liquid suspension, the bubbles adhere to the particles to be separated carrying them at the top of the separator where they are collected (Pragya et al., 2013). Apart from microalgae harvesting, flotation is potentially applied in other fields for the recovery of valuable end-products such as oils (Al-Shamrani et al., 2002; Li et al., 2007; Hanotu et al., 2013), proteins (Aksay and Mazza, 2007; Jiang et al., 2011; Liu et al., 2013b), water and wastewaters remediation (Christenson and Sims, 2011). **Table 5** presents the performance and energy requirement of some reported flotation systems. Even though flotation is believed favorable for microalgae harvesting, there is a number of limitations associated with this technology including the use of surfactants or collectors at different dosage to improve the performance, which requires additional separation units and thus subsequently increasing the process cost. More studies in this area are still needed such as the use of natural surfactants or collectors, and non-consumable electrodes materials (for electroflotation systems) in order to render flotation processes more applicable in commercial microalgae harvesting.

**Table 5:** The performance and energy input assessment of microalgae dewatering by flotation.

Mode of separation	Process performance	Energy (kWh/m^3^)	Reference
Foam flotation	Total suspended solids (%) in concentrate: 1.4–2.4	0.015	Coward et al., 2013
Foam fractionation	Harvesting efficiency: >90%	Not determined	Csordas and Wang, 2004
Dissolved air flotation	Harvesting efficiency: 99.9%	1.5	Weschler et al., 2014
Dissolved air flotation	Total suspended solids (%) in concentrate: 5	7.6	Wiley et al., 2009
Jameson cell flotation	Harvesting efficiency: 97.4%	Not determined	Garg et al., 2014

### Centrifugation

Microalgae centrifugation involves a phase separation of microalgal cells from the suspension by the application of centrifugal force, and it is dependent on the particle size and density of the medium components (Uduman et al., 2010; Rawat et al., 2013). Centrifugation is an advantageous microalgae dewatering technique as it is rapid, easy and effective (Molina Grima et al., 2003). However, the exposure of microalgae cells to high gravitational and shear forces can lead to cell disruption and structural damage (Knuckey et al., 2006), and considering the processing of large volumes of microalgal cultures combined with increased energy consumption, centrifugation process is time consuming and economically unattractive (Rawat et al., 2013). Different centrifugation systems have been reported for microalgae dewatering (**Table 6**).

**Table 6:** The performance and energy input assessment of microalgae dewatering by centrifugation.

Centrifuge	Process performance	Energy (kWh/m^3^)	Reference
Self-cleaning plate separator	Algal recovery efficiency: 95%	1	Weschler et al., 2014
Self-cleaning centrifuge	Total suspended solids (%) in concentrate: 12	1	Molina Grima et al., 2003
Nozzle discharge centrifuge	Total suspended solids (%) in concentrate: 2–15	0.9	Coward et al., 2013
Decanter bowl centrifuge	Total suspended solids (%) in concentrate: 22	8	Coward et al., 2013
Hydrocyclone	Total suspended solids (%) in concentrate: 0.4	0.3	Coward et al., 2013

### Bioflocculation

Bioflocculation refers to naturally induced flocculation due to secreted biopolymers of either the microalgal or bacterial cells. Microalgae dewatering costs could be greatly reduced with bioflocculation because no chemical costs are incurred with little to no energy consumption (Christenson and Sims, 2011). **Table 7** presents the performances of some reported microalgae bioflocculation methodologies. The addition of bioflocculants or bacterial microorganisms that naturally produce flocculants, to a culture of microalgae has been shown to enhance bioflocculation processes and the harvesting efficiency of multiple microalgal species (Christenson and Sims, 2011). Some fungi, for instance, have positively charged hyphae that can interact with the negatively charged microalgal cell surface and cause flocculation (Su et al., 2011; Van Den Hende et al., 2011; Zhang and Hu, 2012; Zhou et al., 2012). The dewatering of microalgae with bioflocculation using bacteria or fungi as a flocculating agent in co-cultivation with microalgae presents the main drawback of microbiological contamination, and possible interference with food or feedstock applications of the microalgal biomass. Naturally occurring microbial flocculants have been used to harvest microalgae for aquaculture and biodiesel production because of their high harvesting efficiency and biodegradability (Oh et al., 2001; Manheim and Nelson, 2013).

**Table 7:** The performance of microalgae dewatering by bioflocculation systems.

Mode of bioflocculation	Harvesting efficiency (%)	Reference
γ-PGA broth bioflocculant	99	Ndikubwimana et al., 2014
Commercial γ-PGA bioflocculant	90–95	Zheng et al., 2012
Bioflocculant from *Paenibacillus* sp. AM49	83	Oh et al., 2001
Co-cultivation of microalgae with filamentous fungi	99	Xie et al., 2013
Fungal pelletization-assisted bioflocculation technology	100	Zhou et al., 2012

## Materials and Methods

### Life Cycle Assessment

By definition, LCA means a systematic environmental management tool used to assess the environmental factors associated with the product system through its life cycle stages, and projecting the environmental performance based on selected functional value of the products (Gnansounou et al., 2009; Khoo et al., 2010, 2011). The assessment takes into consideration the relevant inputs and outputs of a system and evaluates the potential environmental impacts associated with it (Yee et al., 2009; Clarens et al., 2010; Sanz Requena et al., 2011). The primary focus of the LCA investigation may be on the energy demands (Jorquera et al., 2010) and/or CO_2_ emissions of the process chain (Stephenson et al., 2010), especially when LCA is applied for comparing the bioenergy products. This study was performed based on the principles of ISO 14040 (International Standardization Organization [ISO], 2006). The LCA model was utilized for energy input and CO_2_ emissions assessment during microalgae dewatering stages considered in this study.

### Goal and Scope

The general goal of this study is to compare life cycle energy and life cycle CO_2_ of different dewatering technologies for potential application at industrial level of algal biofuel production. Different dewatering technologies and scenarios are evaluated and compared for the development of a most economical, with least energy consumption and low emissions two-stage microalgae dewatering system. The process energy for microalgae dewatering includes energy used directly by the dewatering technology (e.g., for mixing), raw materials (e.g., flocculants or electrode materials), but exclude other form of energy associated with other process units as would be included in the traditional cradle-to-grave life cycle assessment (LCA). The process energy input and CO_2_ emissions during microalgae dewatering stage was the only elements considered in analysis. The scope of analysis was limited in this regard to focus mainly on the unit processes used for microalgae dewatering and avoid the uncertainty associated with upstream and downstream process options. The information provided by this study will serve for full LCA study.

**FIGURE 1:**
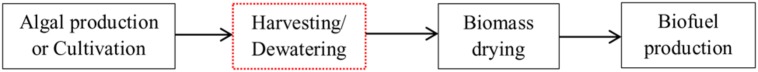
**General flow diagram of algae biofuel production phases**.

The functional unit selected for all dewatering scenarios evaluated is 1000 kg of biomass harvested. **Figure 1** presents a general overview flow diagram of algae biofuel production phases and highlights (with red dashed line) the system boundary of the phase considered in this work. The comprehensive energy input and CO_2_ emissions of different dewatering techniques will assist in finding the most efficient, economic, and environmental friendly two-stage microalgae dewatering configuration. The algal cultivation is not considered in this study; also the drying stage is omitted as some alternative downstream production methods such as wet lipid extraction may be applied.

#### Energy Balance

As harvesting/dewatering is the main bottleneck for the commercialization of microalgae based biofuel due to its high cost resulting from high energy input, it is the stage considered in this work. The volume (*V*) of the microalgae to be processed, the energy input (*EI*) of a single unit dewatering process, total energy input (*TEI*) of the whole dewtering system, were calculated using Eqs (1), (2), and (3), respectively.

(1) $$\begin{array}{cc} V_{x}=\frac{FU}{C_{x}\times RE} & \end{array}$$

(2) $$\begin{array}{cc} {EI}_{x}=V_{x}\times E_{x} & \end{array}$$

(3) $$\begin{array}{cc} {TEI}_{x}={\Sigma EI}_{x} & \end{array}$$

where, *TEI:* Total energy input (kWh/1000 kg dry microalgae), *EI_x_*: Energy input for unit process *x* (kWh/1000 kg dry microalgae), *x*: Unit process, where 1 = first stage dewatering, 2 = second stage dewatering, *V_x_*: Volume of slurry (for *x* = 1, 2) in unit process *x* (m^3^), *E_x_*: Volumetric energy consumption for unit process *x* (kWh/m^3^), *FU*: Functional unit (1000 kg of biomass recovered), *RE*: Recovery efficiency and *C_x_:* Concentration of slurry entering unit process *x*. It is assumed that there is no biomass lost during the downstream process.

#### The CO_2_ Balance

The greenhouse gas (GHG) emissions contributing most apparently to global warming are CO_2_, CH_4_, and N_2_O (Houghton et al., 2001), whereby methane contributes almost 7%, N_2_O about 0.8%, and CO_2_ emissions make up the rest (Dones et al., 2003). Most of LCA studies describe the CO_2_ balance considering the total emissions from fossil fuels and resources consumption vs. the CO_2_ intake by microalgae in cultivation stage. However in this study, the CO_2_ balance is described as the total CO_2_ emitted during dewatering stage based on the amount of the energy required for each process.

#### System Boundaries

To facilitate the development of the analytical framework of the process evaluation, the system boundaries is simulated according to the following conditions and assumptions to typify a large-scale microalgae dewatering systemml:

✓ The case study considered in this work is dewatering stage of a microalgae based biofuel system as presented in **Figure 1**.
✓ As this is a comparative operational life cycle analysis, some of the components of the full life cycle analysis framework, such as energy production and distribution, for the different technologies will cancel out. Thus the main LCA driving component is the energy consumption/requirement of the different dewatering technologies.
✓ The functional unit which is the measure of the performance and functional output of the system is chosen as 1000 kg of microalgae biomass in the final slurry.
✓ The initial microalgae biomass concentration is 0.3 g/L, which reflect the biomass concentration in open pond cultivation systems.
✓ The electricity consumed during the process is generated from black coal with associated environmental impacts (Norgate and Rankin, 2001).
✓ The amount of CO_2_ emitted was calculated based on the amount of energy required for each process.
✓ The operational cost calculated includes the aluminum cost and nano particles costs.
✓ The leakage or malfunction of the system is not taken into consideration.
✓ The emissions of wastewater and other air pollutants are not covered in this study.
✓ The treatment of wastes (such as solids or wastewater) is not considered in this study.

### Process Development and Evaluation of Two-Step Microalgae Dewatering Techniques

**FIGURE 2:**
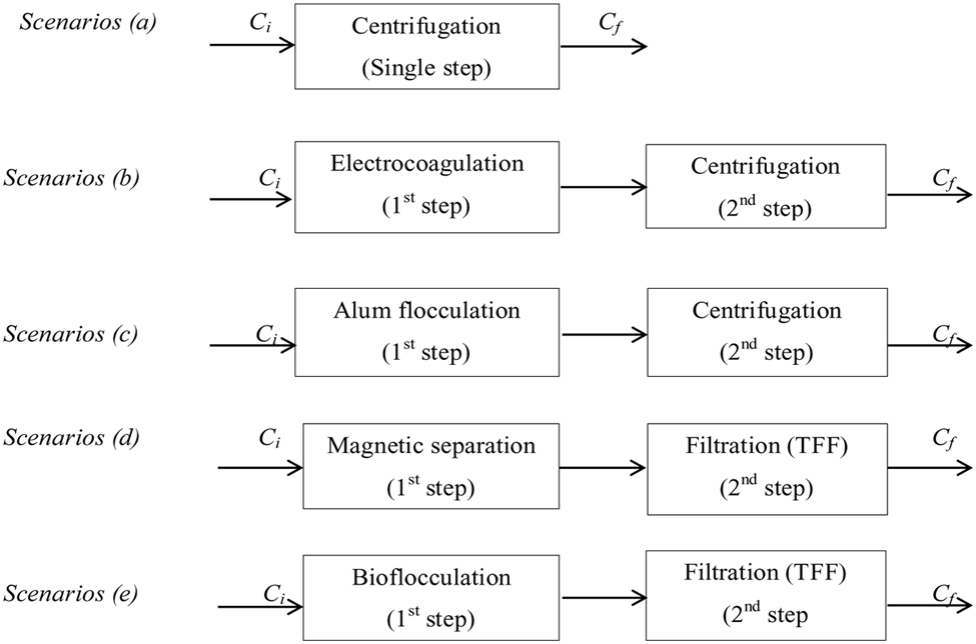
**Block diagram of one-step (a) and two-step (b–e) microalgae dewatering scenarios (*C_i_* and *C_f_* initial and final microalgae concentration, respectively)**.

Most existing microalgal biomass production systems use energy intensive centrifuges for harvesting and dewatering microalgae (Heasman et al., 2000), making harvesting and dewatering a major fraction of the total energy demand (Molina Grima et al., 2003; Uduman et al., 2010). The application of two-stage dewatering techniques has been found to significantly improve the process (Can et al., 2006). LCA is important to assess the environmental aspects and potential impacts associated with the technology. Classical LCA implements a ‘cradle to grave’ principle which investigates the environmental aspects throughout the life of the product; raw material acquisition, product manufacturing, product use and disposal. The two-step microalgae dewatering techniques proposed for evaluation are presented in **Figure 2** as follows: (b) electro-coagulation with aluminum anodes coupled with centrifugation, (c) alum flocculation coupled with centrifugation, (d) magnetic separation coupled with TFF, and (e) bioflocculation coupled with filtration. Centrifugation (a) is used as the single-step dewatering base scenario for the analysis since it is the most conventional method used in the industry. All dewatering systems are assumed to be operated in batch mode. Considering different existing microalgae dewatering techniques, it is possible to have several various combinations in two-step system. In this study, only four combinations were proposed and further studies can evaluate other combinations.

For the electrocoagulation, aluminum anodes were chosen over steel 430 anodes as coagulation rate is faster with aluminim anodes, maximum recovery of microalgae is obtained at shorter run times, and smaller quantities dissolved aluminim is required under the same electro-coagulation conditions (Uduman et al., 2011). Moreover, Vandamme et al. (2011), realized that ECF was more efficient with aluminum anode than with iron anode. For the purpose of this study, aluminum metal dissolution and requirement are determined based on the batch analysis, alum flocculation dosage is determined from batch experiments, and the electrocoagulation energy requirement is based on optimal reported conditions (Uduman et al., 2011). Reported LCA and experimental data are used in this study to determine the energy requirements, carbon dioxide emissions, and cost for the proposed dewatering techniques.

Parameters and data sources essential to carry out this analysis are summarized in **Table 8.** The data presented in **Table 8** are the representative averages of different reported values of the same unit operation. Other sources of data are: Fe_3_O_4_ nano particles price: $ 114.2/kg (Alibaba/Beijing Dekedaoin Tech. Co. Ltd, 2014), CO_2_ emission rate: 1.115 kg/kWh (Dones et al., 2003), Energy to produce Aluminumml: 58.61 kWh/kg (Norgate and Rankin, 2001), Energy to produce alumml: 3.06 × 10^–4^ kWh/kg (Arpke and Hutzler, 2006), the cost of aluminum anodes, flocculants and magnetic nano particles were also included in cost calculations. Aluminum cost was taken to be $ 1.733/kg^1^, alum cost $ 0.2/kg^2^, the electricity cost was $ 0.105/kWh (The State Grid Corporation of China/Fujian Province (2012), The industry power price), and the industrial price of Fe_3_O_4_ nano particle, size of 20 nm (99% of purity) was 700 RMB/kg (Alibaba/Beijing Dekedaoin Tech. Co. Ltd, 2014). The exchange rate was: 100USD = 613.2RMB^3^ Aluminum and alum production energy requirements were included in calculations of energy inputs for electrocoagulation and alum flocculation, respectively.

**Table 8:** Data sources for energy input, carbon dioxide emission, and cost analysis.

Unit	Microalgae recovery (%)	Energy (kWh/m^3^)	Reference
Self-cleaning plate centrifuge	95	1	Weschler et al., 2014
Electrocoagulation	88	0.6	Vandamme et al., 2011
Flocculation	99	0.1	Weschler et al., 2014
Magnetic separation	98	Not determined^∗^	Xu et al., 2011
TFF	89	0.2	Weschler et al., 2014
Bioflocculation	99	Negligible	Xie et al., 2013

^∗^*The energy input required for magnetic separation will be calculated in this study based on mixing energy.*

The mixing energy required for magnetic separation was calculated by determining the mixing power consumption of the stirrer blades for the total mixing time (Sinnott, 2005). The shaft power required to drive an agitator can be estimated using the power number:

(4) $$\begin{array}{cc} N_{P}=\frac{P}{D^{5}N^{3}\rho } & \end{array}$$

Where *D* is the agitator diameter (*m*), *N* is the agitator speed (s^–1^), ρ is the fluid density (kg/m^3^), and *P* is the shaft power (w). Equation (4) can be rearranged to solve for the shaft power. The power number can be obtained by using the power correlation for a single three bladed propeller and the Reynolds number (Re).

(5) $$\begin{array}{cc} R_{e}=\frac{D^{2}N\rho }{\mu } & \end{array}$$

Where μ is the fluid viscosity (Ns/m^2^).

## Results And Discussion Of Two-Stage Dewatering Systems

**FIGURE 3:**
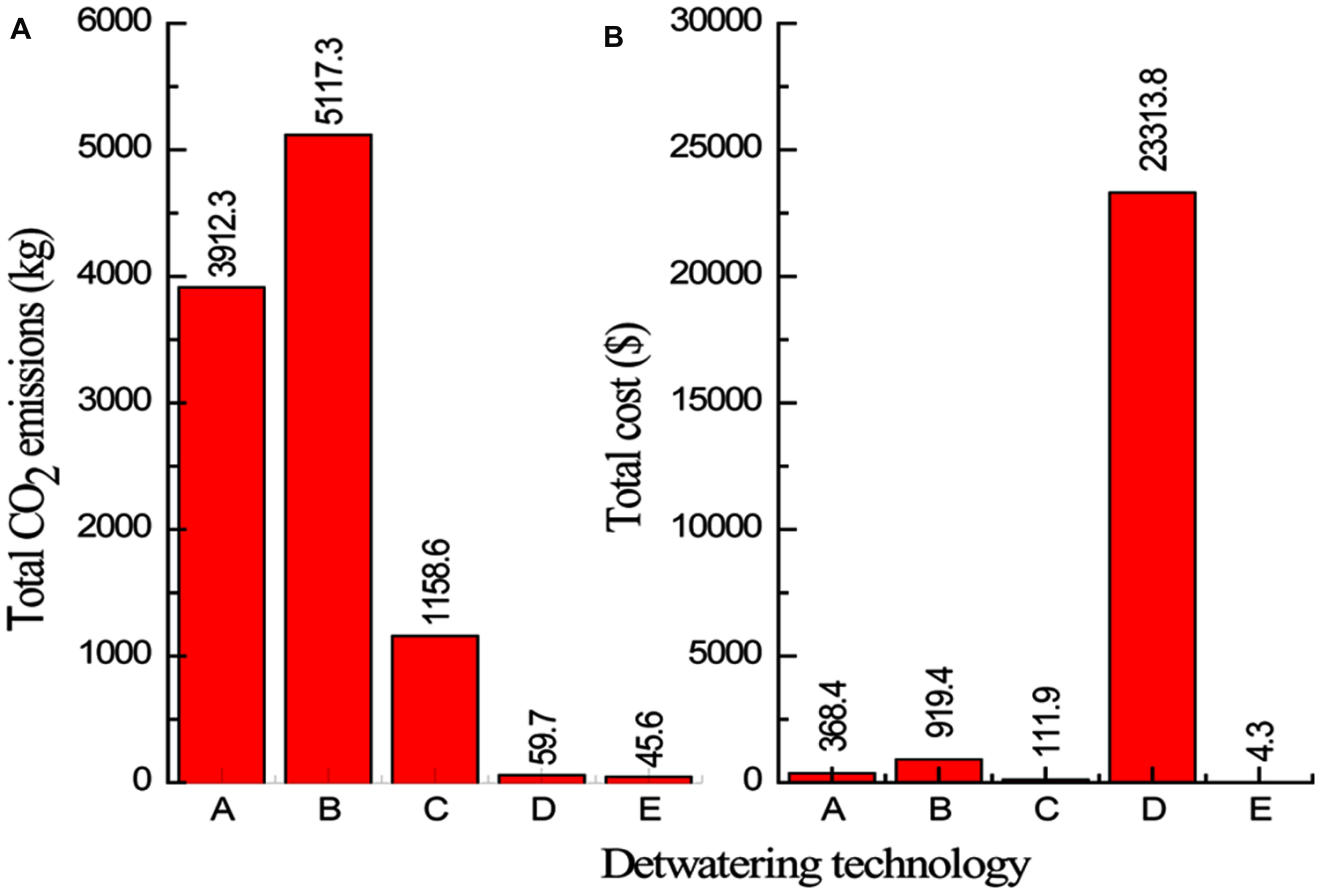
**Total CO_2_ emissions **(A)** and total cost **(B)** for the dewatering technologies evaluated**.

After LCA calculations and process cost evaluation, the total energy demands, CO_2_ emissions and the costs for the different scenarios of microalgae dewatering are presented in **Table 9** and **Figure 3.**

**Table 9:** Energy requirements for the dewatering technologies evaluated.

Unity process		Energy input (kWh)
Centrifuge	Centrifugation	3508.8
Electrocoagulation/Centrifuge	Al production	1722.2
	Electrocoagulation	2272.7
	Centrifugation	594.5
	**Total**	4589.4
Alum flocculation/Centrifuge	Alum production	0.6
	Alum flocculation	336.7
	Centrifugation	701.8
	**Total**	1039.1
Magnetic separation/TFF	Magnetic separation	0.03
	TFF	53.5
	**Total**	53.5
Bioflocculation/TFF	Bioflocculation	Negligible
	TFF	40.9
	**Total**	40.9

(A: Centrifugation, B: Electrocoagulation coupled with centrifugation, C: Alum flocculation coupled with centrifugation, D: Magnetic separation coupled with Tangential flow filtration (TFF), and E: Bioflocculation coupled with filtration).

The two-step dewatering system of electrocoagulation coupled with centrifugation emerged the most energy intensive (**Table 9**), with highest CO_2_ emissions but with relatively low cost (**Figure 3**). Different studies conducted on electrocoagulation show that it is a promising microalgae dewatering technique due to its lower energy demand. Vandamme et al. (2011) found that under optimal conditions, power consumption of Electrocoagulation–Flocculation using aluminum anode, was around 2 kWh/kg of microalgal biomass harvested for *C. vulgaris* and 0.3 kWh/kg for *P. tricornutum*, thus authors concluded that ECF is more energy efficient compared to centrifugation. In their study on the removal of COD from wastewater with alum flocculation and electrocoagulation, Can et al. (2006), reported that energy input of 1.2 kWh/kg COD and an operating cost of $ 0.31/kg COD were necessary for electrocoagulation, while energy requirement of 0.02 kWh/kg COD and an operating cost of $ 0.08/kg COD are for alum flocculation. However, these studies only accounted for the energy and costs associated with the operation and did not consider the energy, CO_2_ emissions and costs associated with raw material production which is an important parameter to consider for a complete life cycle assessment (LCA).

The two-step dewatering systems of magnetic separation and bioflocculation pared with TFF are almost in the same range of energy input (**Table 9**), and CO_2_ emission (**Figure 3**), per 1000 kg of microalgae biomass recovered, however, the system of magnetic separation coupled with TFF is the most costing compared to all other microalgae dewatering systems evaluated (**Figure 3**). This is due to the high cost of magnetic nano particles. The recycle and reusability frequency of nano particles should be evaluated in order to cut-down the cost of magnetic separation process. Xu et al. (2011), reported that magnetic separation was energy-saving and environmental friendly microalgae dewatering technique but authors did not consider the process cost effectiveness because the cost of magnetic nano particles was not included in the study. Therefore, for the system of magnetic separation coupled with TFF to be cost effective, the use of cheaper and reusable magnetic nano particles is imperative. From the analysis, the two-step microalgae dewatering by bioflocculation pared with TFF process is the most attractive in terms of energy consumption, CO_2_ emissions, and cost, showing significant low values for each parameter compared to centrifugation as a single step and other two-step dewatering systems considered in this study. However, initial capital investment for TFF is greater than flocculation as reported by Danquah et al. (2009a), but the payback period is 1.5 years earlier than 6 years for flocculation thus greater profits can be obtained with TFF, therefore this renders the two-step microalgae dewatering technique of bioflocculation coupled with TFF a viable, effective and cost efficient microalgae dewatering technique that can be applied at industrial level. Previous alum flocculation studies have deemed it unsuitable for microalgae dewatering due to high dosage requirement (Buelna et al., 1990). However, from their studies on alum flocculation of marine microalgae, Uduman et al. (2011), reported that the alum dosage was in moderate quantities for commercial considerations thus making alum flocculation a possible industrial microalgae dewatering technique. Moreover, the coupling of alum flocculation with centrifugation in a two-step microalgae dewatering process for microalgae biomass production is profitable with the lowest energy input and CO_2_ emissions compared to centrifugation as a single step and electrocoagulation coupled with centrifugation dewatering processes.

The results from this study demonstrate that a two-step microalgae dewatering technique, though it requires a high initial capital investment, is more efficient, economic viable and environmental friendly compared to a single step. Also Can et al. (2006), and Sander and Murthy (2010), reported that two-step dewatering technique is more energy efficient compared to a single step. Bilad et al. (2012), reported that the energy consumption to dewater *C. vulgaris* and *P. tricornutum* was 0.84 and 0.91 kWh/m^3^, respectively, when the submerged microfiltration was combined with centrifugation in a two-step dewatering system. However, the results from the present study clearly show that bioflocculation coupled with TFF would be the best choice for microalgae dewatering with the lowest energy consumption. Different LCA studies have also confirmed that the two-step microalgae dewatering is the most efficient with low energy consumption and low emissions. Weschler et al. (2014) compared the process energy of different cultivation and harvesting technologies combined in various production scenarios for the production of microalgae biomass as a biofuel feedstock. Authors realized that the scenarios which used open ponds for cultivation, followed by settling and membrane filtration were the most energy efficient. Khoo et al. (2011), during their study on life cycle energy and CO_2_ analysis of microalgae to biodiesel, they employed air sparging assisted coagulation flocculation (ASACF) process followed by centrifugation for harvesting and dewatering. They found that on the basis of a functional unity of 1 MJ of biofuel, the total energy demands were 4.44 MJ with 13% from biomass production, 85% from lipid extraction, and 2% from biodiesel production. O’Connell et al. (2013) reported the LCA of dewatering routes for algae derived biodiesel processes. Authors performed an analysis of the life cycle emissions associated with harvesting, dewatering, extraction, reaction, and product purification stages for algae biodiesel. From this base-case, they found that the total emissions were 10,500 kg per 1 t of biodiesel with 96% of those attributed to the spray dryer used for dewatering. However, by evaluating different alternative cases for various sequences of mechanical and thermal dewatering techniques, authors realized that the best case consisted of a disk-stack centrifuge followed by the chamber filter press and a heat integrated dryer, which resulted in 875 kg emissions per 1 t of biodiesel, equivalent to a 91% reduction from the base-case.

## Conclusion

This study provides viable information about the energy input, environmental impact, and cost related to a two-step microalgae dewatering technique compared to a single step. The information provided by this study will contribute much essentially for a full LCA of a microalgae based biofuel process. The comparison of the two-step dewatering processes proposed in this study, and centrifugation as a single dewatering technique demonstrated that two-step dewatering technique can be less energy intensive, with low CO_2_ emissions, cost efficiency, and high microalgae recovery. Life cycle energy analysis and related carbon dioxide emission revealed that bioflocculation coupled with TFF is the most promising industrial microalgae dewatering technique having the lowest energy consumption and carbon dioxide emissions. Magnetic separation required low energy for its operation as a dewatering process, but the overall process was highly costing, due to the cost associated with the Fe_3_O_4_ nano particles. Even though, a two-step microalgae dewatering technique is promising, more work is required to investigate process conditions required for optimal recovery at low energy requirement, carbon emission and overall cost of the dewatering process for a complete LCA study. Moreover, to minimize the production cost of biofuels from microalgae, extensive studies including strains engineering as the dewatering might be strain dependent, further combination of other different single dewatering technologies, optimization of different working parameters, choice of process materials and chemicals, and rigorous life cycle assessments are required to develop an efficient process for large-scale microalgae dewatering.

## Author Contributions

MKD, XZ, conceived, designed and supervised the study with the help of YL and LL. RRS and TN collected the sources data and wrote the draft of the manuscript. All authors revised the manuscript and contributed to the discussion and approved the final manuscript.

## Conflict of Interest Statement

The authors declare that the research was conducted in the absence of any commercial or financial relationships that could be construed as a potential conflict of interest.

## Footnotes

1 http://www.alibaba.com/product-detail/2012-August-Price-for-Ammonium-Alum622064205.html

2 http://www.metalprices.com/metal/aluminum/lme-aluminum-cash-official, 2014

3 http://www.boc.cn/sourcedb/whpj/.
